# Physical activity and sedentary behaviour amongst children with obesity - exploring cross-sectional associations between child and parent

**DOI:** 10.1186/s44167-025-00072-0

**Published:** 2025-02-13

**Authors:** Hannah Lundh, Daniel Arvidsson, Christian Greven, Jonatan Fridolfsson, Mats Börjesson, Charlotte Boman, Katarina Lauruschkus, Stefan Lundqvist, Karin Melin, Susanne Bernhardsson

**Affiliations:** 1 Region Västra Götaland, Centre for Physical Activity, Gothenburg, Sweden; 2 School of Public Health and Community Medicine, Institute of Medicine, Sahlgrenska Academy, University of Gothenburg, Gothenburg, Sweden; 3 Center for Health and Performance, Department of Food and Nutrition, and Sport Science, University of Gothenburg, Gothenburg, Sweden; 4 Center for Lifestyle Intervention, Dept of Molecular and Acute Medicine, Sahlgrenska Academy, University of Gothenburg, Gothenburg, Sweden; 5 Region Västra Götaland, Dept of MGAÖ, Sahlgrenska University Hospital, Gothenburg, Sweden; 6 Faculty of Medicine, Department of Health Sciences, Lund University, Lund, Sweden; 7 Department of Habilitation, Committee on Psychiatry, Habilitation and Technical Aids, Region Skåne, Malmö, Sweden; 8 Region Västra Götaland, Research, Education, Development and Innovation, Primary Health Care, Gothenburg, Sweden; 9 Unit of Physiotherapy, Department of Health and Rehabilitation, Institute of Neuroscience and Physiology, Sahlgrenska Academy, University of Gothenburg, Gothenburg, Sweden; 10 Gillberg Neuropsychiatry Centre, Institute of Neuroscience and Physiology, Sahlgrenska Academy, University of Gothenburg, Gothenburg, Sweden; 11 Region Västra Götaland, Department of Child and Adolescent Psychiatry, Sahlgrenska University Hospital, Gothenburg, Sweden

**Keywords:** Accelerometer, Physical activity, Sedentary time, Children, Obesity

## Abstract

**Background:**

Physical activity (PA) in childhood is critical for establishing a healthy lifestyle across the lifespan, particularly to treat and prevent obesity. This study aimed to explore PA and sedentary behaviour (SED) in 6–12-year-old children with obesity and their parents, and possible associations in these behaviours between children and parents.

**Methods:**

Children referred to outpatient paediatric healthcare for obesity treatment and one of their parents wore accelerometers (Axivity) on their hip during seven consecutive days. Accelerometer data were processed using the 10 Hz frequency extended method. Correlations between child and parent PA and SED, respectively, were analysed using intra-class correlation coefficient.

**Results:**

Thirty-nine children (19 female) and 38 parents (20 female) were included. The mean age of the children was 9.7 years (SD 2.0) and the mean parent age was 42.2 years (SD 6.1). The mean child BMI-SDS was 3.0 (SD 0.4). Fifty-seven % of the parents were born in Sweden, 16% in other European countries, and 27% outside Europe. Children spent an average of 9.8 h/day in SED, while parents spent an average of 12.3 h/day. The mean daily time spent in low-intensity PA was 3.9 h for children and 3.4 h for parents, while moderate-to-vigorous intensity PA averaged 0.7 h/day for children and 0.3 h/day for parents. Only six of the children (15%) reached the recommended minimum of 60 min of moderate-to-vigorous intensity PA per day and only two parents (5%) reached the recommended weekly minimum of 150 min of moderate intensity PA. Child and parent SED was significantly correlated, although the correlation was weak (ICC 0.14; p = 0.017). No statistically significant correlations were found for any of the analysed PA intensity levels.

**Conclusions:**

The findings indicate an association between children’s and parents’ SED in this sample of school-aged children with obesity, while no association was observed in PA behaviour. Generalisability of our findings is limited and more research is needed– in larger samples, other settings, and using longitudinal designs– to better understand the potential links between the PA patterns of children with obesity and that of their parents.

**Supplementary Information:**

The online version contains supplementary material available at 10.1186/s44167-025-00072-0.

## Background

Obesity is a public health issue of global concern, a major risk factor for non-communicable diseases, and responsible for a large part of disease burden worldwide [1]. Prevalence of obesity amongst adults, children and adolescents has increased globally during the last decades [2]. Children are especially vulnerable since obesity in young age tend to track into adulthood [3, 4]. In 2019, 21% of Swedish children aged 6–9 had overweight or obesity, of whom 6% had obesity [5]. With the COVID-19 pandemic reducing physical activity (PA) and increasing sedentary time (SED), several countries reported increased weight gain [6]. Children with obesity suffer from increased risk of health conditions similar to those seen amongst adults with obesity, i.e., complications such as endocrine, metabolic, respiratory, cardiovascular, and psychosocial health issues [6–8]. Tracking of obesity into adulthood results in further health risks; for example, different cancers [6, 9] and premature mortality [10].

The underlying factors causing obesity are complex and multifactorial [7, 11]. On an individual level genetic pre-disposition contributes [7, 11], while a poor diet and physical inactivity are main drivers of weight gain [12]. Physical activity has well-established positive health effects for all children [13]. Beneficial effects of PA on cardiovascular health have been reported especially for children and adolescents with overweight/obesity [14, 15]. For treatment of childhood obesity, WHO recommends family-based interventions that are delivered by multi-professional teams, and include PA, nutrition, and psychosocial support [11]. Promoting healthy behaviours through family-based behaviour change strategies to achieve healthy nutritional and PA habits are considered first-line treatment [6, 7]. However, there is a lack of effective interventions for children with obesity and a need for more research on key components of multicomponent interventions and optimal ways to involve parents [16]. Parents/guardians (hereinafter parents) could function as enablers for children’s PA in different ways [17, 18], and family-centredness and parental involvement are at the core of obesity treatment and PA interventions for children [19].

Given the importance of the family context for children’s health behaviours, parents are likely to be accessible role models for their children and their health behaviours are observed by their children. If parents contribute to children’s PA by being role models through their own PA, there should presumably be an observable association between parent and child PA. Reviews of studies on predominantly healthy, non-obese, children indicate that child and parent PA are associated [20, 21], which could have implications for parental involvement in interventions aiming at increasing children’s PA. Only one of the 65 studies included in the two reviews examined child-parent associations in an obese paediatric population using accelerometry, also showing an association between child and parent PA [22]. The variety of methods used for measuring PA makes it difficult to compare studies [23, 24], and research on accelerometer-measured PA amongst children with obesity and their parents is very limited. Children with obesity are less physically active than their non-obese counterparts [25]. Parents of children with obesity are also less physically active than parents of normal-weight children, likely influencing their children’s PA levels [26]. Parents of children with obesity have different perceptions of their child’s weight and PA needs than parents of normal-weight children, and are less likely to recognise the weight status of their children and its potential health consequences [27, 28]. They might underestimate the importance of PA or overestimate their child’s activity levels [29], which could lead to less encouragement and support for PA compared to parents of normal-weight children. Furthermore, neurodevelopmental disorders are more prevalent amongst families with obesity, which may influence parents’ ability to support their children’s PA due to adaptations already made to accommodate the neurodevelopmental condition [30, 31]. As physical inactivity is an important contributor to maintaining childhood obesity, further research on PA patterns among children with obesity and their parents is needed. This population might exhibit unique dynamics in this relationship; parents may also be obese and physically inactive, and model a sedentary lifestyle that might be mirrored by their child [26]. Therefore, the aim of this study was to explore accelerometer-measured PA and SED in a Swedish sample of 6–12-year-old children with obesity and their parents, and possible associations between child and parent PA and SED.

## Methods

### Study design

This study is part of the research project “Implementation of physical activity on prescription for children with obesity in paediatric health care (IMPA)”, aiming to investigate the feasibility of implementing physical activity on prescription (PAP) for children with obesity in paediatric health care [32]. The study employed a cross-sectional design and examined PA and SED of children and parents at baseline, before participants received PAP treatment. The study is reported in accordance with the STROBE guideline (Additional file 1).

### Setting and participants

All paediatric outpatient clinics in Sweden’s second largest city, Gothenburg, and surrounding municipalities in Region Västra Götaland (totally 12 clinics) were invited to participate. Senior management approved of the study and initially selected three clinics in which the study could be conducted: two specialist centres for children and youth in Gothenburg and one paediatric outpatient clinic in the adjacent municipality of Mölndal. After the first study year, two more paediatric clinics in the municipalities of Alingsås and Lerum and one rehabilitation clinic in Gothenburg were appointed. The paediatric clinics all offer treatment for children with obesity in multi-professional teams typically including a paediatrician, a nurse, a dietician, and a psychologist. The appointed rehabilitation clinic has a close collaboration with a paediatric clinic in Gothenburg. Within the IMPA project, healthcare professionals working in the teams received training in recruitment, effects of PA, the intervention, and the digital application used to collect data.

### Data collection

Participants were recruited between January 2022 and May 2024. Inclusion criteria were: age 6–12, diagnosed with obesity (BMI > ISO-BMI 30), and insufficient PA level according to Swedish national recommendations [13]. Insufficient PA level was assessed based on the child’s and parent’s estimate of the child’s PA during the last week. Exclusion criteria were: severe psychiatric comorbidity, severe intellectual or physical disability, or planning to relocate outside the study area within 12 months.

Data on PA and SED were collected using waist-worn accelerometers (Axivity AX3, Axivity Ltd, UK) placed over the participant’s hip. The placement, close to the body’s centre of mass, was chosen because it is the most suitable position to capture whole-body activity [33], which was the main focus of this study. Accelerometer sampling rate was set to 50 Hz and an acceleration range expressed in gravity (g) of ± 8 g. Children and parents were instructed to wear the accelerometer in an elastic belt around the waist, above the right hip, 24 h per day, for seven consecutive days. It could be worn outside or inside their clothes, at their discretion. Wearing the belt during sleep was recommended but not mandatory; while the study did not intend to measure sleep outcomes, wearing the belt during the night was recommended to reduce non-wear time. To help identify sleep and non-wear time, participants registered bedtime, wakeup-time, cycling, water-based activities, and strength training during the 7-day period in an activity diary, on paper or via a project-developed digital communication platform. This platform comprised a patient mobile app and a healthcare practitioner web-based interface, both with secure access. The participants were instructed to fill out the diary each evening before bedtime. Sociodemographic data were collected, either by completing a paper-based questionnaire or by filling out a digital form in the mobile app, on the children’s age, sex, BMI and comorbidity and on parent’s age, sex, place of residence, country of birth, education level, employment, and any diseases.

### Variables

When processing data from accelerometers, a filter is applied to avoid capturing noise not related to PA. An often applied low-pass cut-off filter can misclassify PA data of higher intensities, and a wider filter is needed to better capture actual PA [34, 35]. Raw accelerometer data were therefore processed to a PA intensity measure (mg) using the 10 Hz Frequency Extended Method (FEM), which has been shown to better capture PA at higher intensities [34]. Furthermore, since children’s PA often occur in short bouts, reducing data into a short time window is more appropriate [23], so 3-second epochs were used to better capture variation in PA. For definition of an average week, a valid day was defined as at least 10 h of wear time, and a valid week as at least two valid days. Traditionally, a minimum of four valid days is suggested [23]. However, sensitivity analysis showed no difference between using two versus four valid days, so two valid days were chosen to maximise the sample size. Non-wear time was defined as 60 min of consecutive zero acceleration output, with the allowance of up to two minutes of output below the sedentary threshold [23]. Data were distributed into the crude intensity categories SED, light PA (LPA), and moderate-to-vigorous PA (MVPA). MVPA is the intensity category most important for health outcomes and part of the PA recommendation for children, while guidelines also recommend reducing SED [13, 36]. Cut-points for LPA and MVPA are often defined as above 1.5 METs and 3 METs, respectively, in both children and adults. However, for both children and adults, the traditional cut-point for MVPA at 3 METs is set rather low, corresponding to a walking speed of 3–4 km/h [37] indicating a slow pace [38, 39]. Furthermore, at 3 METs the association with cardiometabolic health is minimal while starting to become clinically relevant from 4 METs, for both children and adults [40, 41]. This level corresponds to a brisk walking pace of 4–5 km/h [37]. We therefore chose to use 4 METs as the cut-point for MVPA in both children and adults. Accelerometer cut-points representing 1.5 and 4 METs (52 mg and 388 mg for children, 39 mg and 331 mg for adults, respectively) were based on a previous calibration study [34].

### Data analysis

Child and parent demographic characteristics are presented as means (SD) or numbers (percentages). Sex- and age-adjusted body mass index (BMI) standard deviation score (BMI-SDS) was calculated using a Swedish reference population from Gothenburg [42]. Means and standard deviations for children’s and parents time spent in SED and different PA intensity levels were computed. Moderate and vigorous intensity were combined since vigorous intensity is often difficult to analyse separately, due to few collected minutes and skewed distribution. Data distribution was explored visually and tested with the Shapiro-Wilk test for normality, guiding our choice of tests. Data were approximatively normally distributed for SED and LPA but were positively skewed for MVPA in both children (*p* = 0.015) and parents (*p* < 0.001). Since a proportion of participants did not adhere to the 24-hour wear protocol and many activity diaries were incomplete, 24-hour analysis including sleep was not performed.

Correlation and test of difference were used for the comparative analyses of SED, LPA and MVPA between child and parent. To capture within-pair correlations for each child-parent pair, we used absolute agreement Intraclass Correlation (ICC), in two-way mixed-effect models and single measures. To interpret the strength of the correlations, we applied the cut-offs suggested by Koo and Li [43], where values under 0.5, between 0.5 and 0.75, between 0.75 and 0.9, and above 0.90 are indicative of weak, moderate, strong, and very strong correlation, respectively. For test of difference, a paired t-test or the Wilcoxon’s signed rank test for paired data was used, respectively, and 95% confidence intervals of the difference were calculated. In addition, Bland-Altman plots were used to visualise the variation in the comparison between child and parent. Statistical analyses were performed in IBM SPSS Statistics (version 28). Statistical significance was set to *p* < 0.05.

### Ethics approval and consent to participate

The study was approved by the Swedish Ethical Review Authority (reference no. 2021–03632, 2021-06810-02). Written and verbal informed consent were collected from participating parents/guardians and verbal assent from the children.

## Results

A total of 102 children met the inclusion criteria and were invited to participate. Forty-two children and 40 parents accepted to participate (two of the parents enrolled two children). Of those, 39 children (19 female) and 38 parents (20 female), i.e., 39 child-parent pairs, provided valid accelerometer data and could be included in the study.

Participant characteristics are presented in Table 1. The children’s mean age was 9.7 (SD 2.0) years, and mean BMI-SDS was 3.0 (SD 4.5). Participating parents’ mean age was 42.2 (SD 6.1) years. 57% of parents were born in Sweden, 16% were born in other European countries, and 27% were of non-European origin. 92% had secondary school or higher; 51% were blue-collar workers and 46% were white-collar workers.

**Table 1:** Characteristics of participating children and parents

Variable	Children (*n* = 39)	Parents (*n* = 38)
Sex^a^:		
Female	19 (48.7)	20 (52.6)
Male	20 (51.3)	18 (47.4)
Age^b^, years	9.7 (2.0)	42.2 (6.1)
BMI^b^, kg/m^2^	25.7 (3.4)	Not collected
BMI-SDS^b^, units	2.96 (0.45)	Not collected
Country of birth^a^:		37
Sweden		21 (56.8)
Europe, excluding Sweden		6 (16.2)
Outside Europe		10 (27.0)
Education^a^:		37
Primary school or less (≤ 9 years)		3 (8.1)
Secondary school or equivalent		21 (56.8)
University		13 (35.1)
Employment^a^:		37
Blue-collar worker		19 (51.4)
White-collar worker		16 (43.2)
Sick leave		2 (5.4)
Comorbidities/other relevant conditions	12* (asthma, allergy, ADHD, autism, gastritis, cerebral palsy)	15** (allergy, diabetes mellitus, hypertension, anxiety, Chron’s disease, Parkinson’s disease, endometriosis)
Neuropsychiatric investigation, ongoing or planned	2*	

ADHD = Attention deficit/hyperactivity disorder; BMI = Body mass index; BMI-SDS = Body mass index-standard deviation score

Values are given as ^a^ number (percentage) or as ^b^ mean (standard deviation)

*Reported by healthcare professional

**Self-reported

**Fig. 1:**
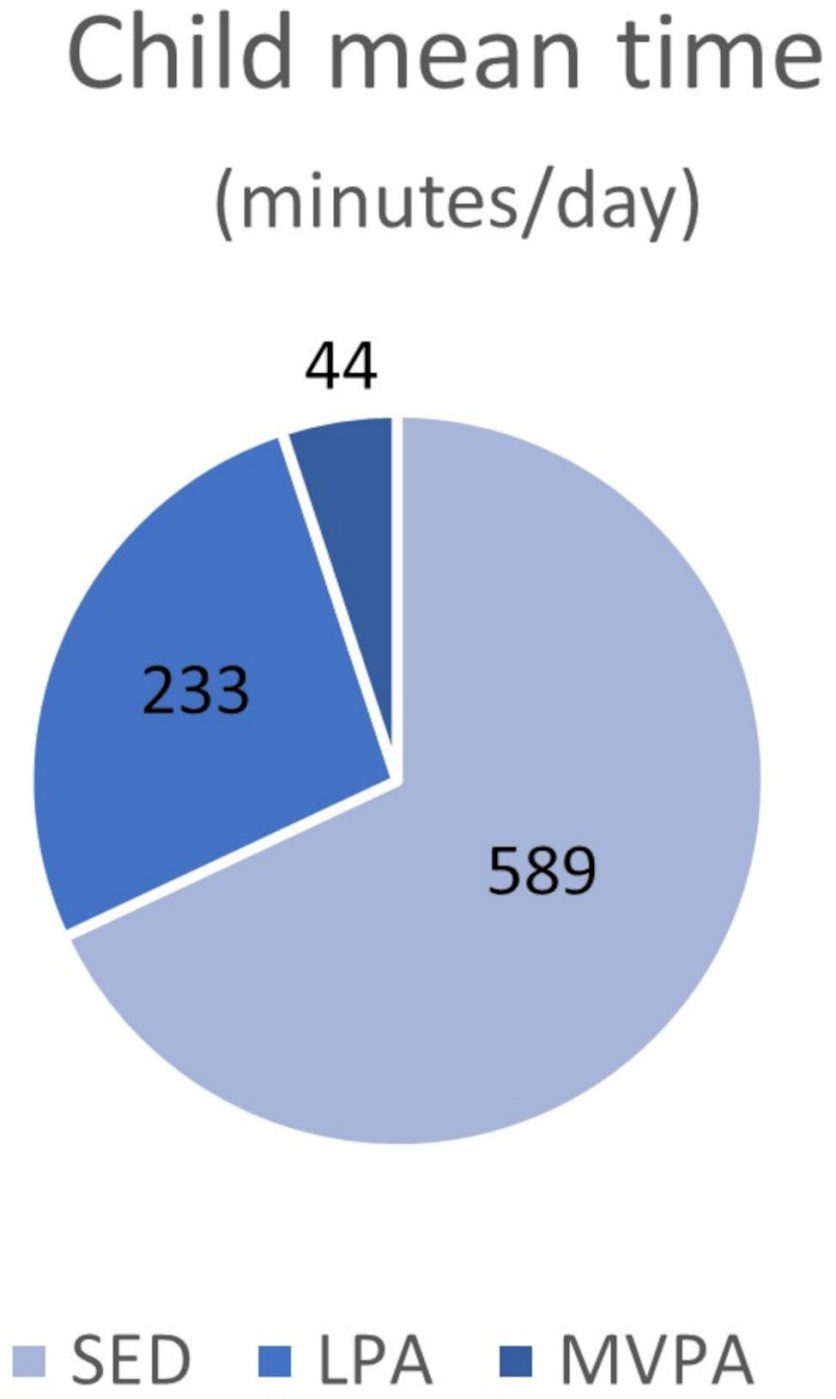
Child mean time spent sedentary (SED), in light physical activity (LPA), and in moderate-to-vigorous physical activity (MVPA)

**Fig. 2:**
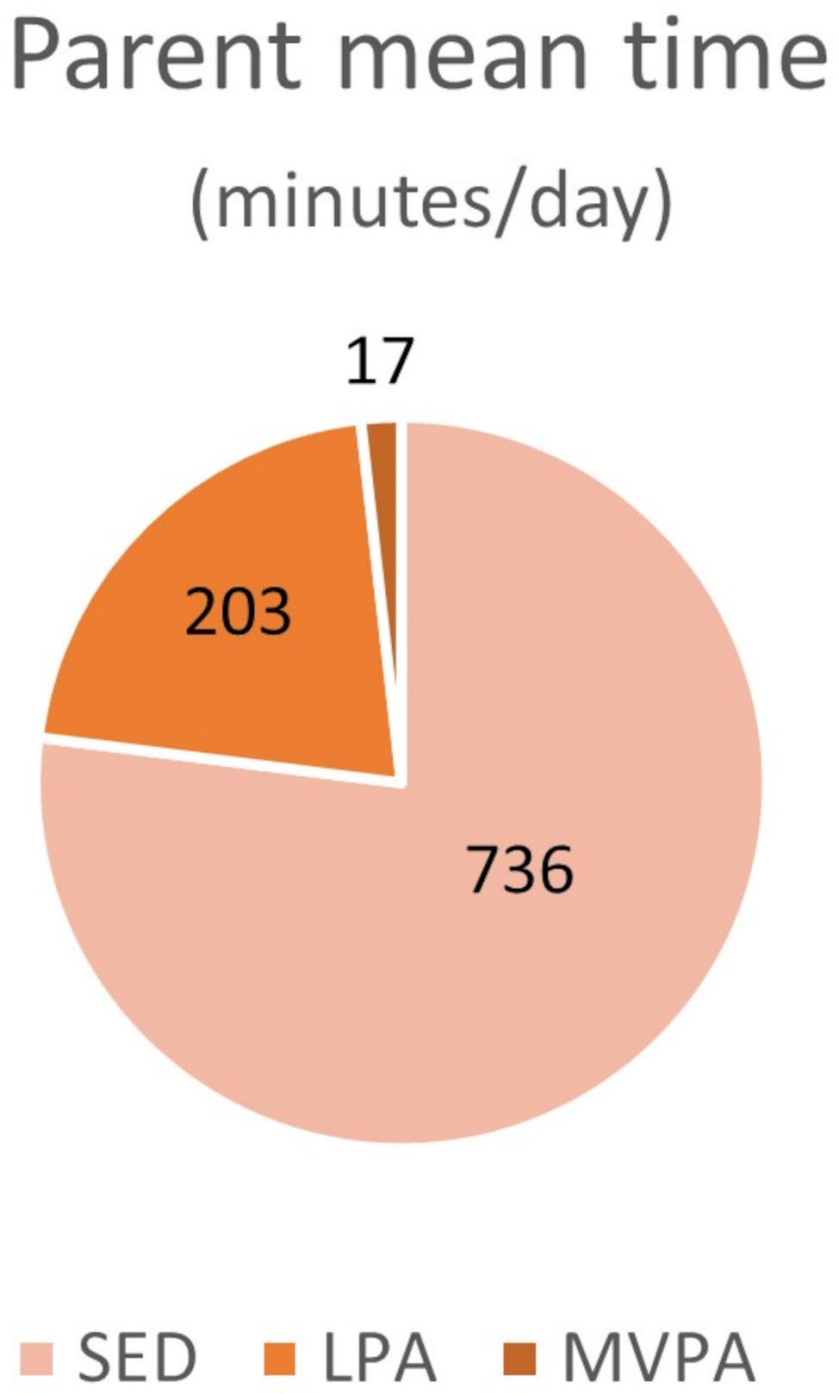
Parent mean time spent sedentary (SED), in light physical activity (LPA) and in moderate-to-vigorous physical activity (MVPA)

Figures 1 and 2 illustrate mean time spent in sedentary and different physical activity categories by the children and their parents.

Means and standard deviations for time spent in SED and the different PA levels, intraclass correlations, and paired differences between children and their parents, are presented in Table 2. A significant, but weak, correlation between child and parent was found for SED only (ICC 0.14, *p* = 0.017). A stronger correlation was observed for LPA, although it did not reach statistical significance (ICC 0.24, *p* = 0.050). For MVPA a weak, non-significant, correlation was found (ICC 0.11, *p* = 0.081). On average, children spent significantly more time engaged in MVPA compared to their parents (mean paired difference approximately 27 min; *p* < 0.001). Parents spent significantly more time in SED than the children (mean 12.3 versus 9.8 h/day; *p* < 0.001). Only six of 39 children (15.4%) reached the recommendation for children aged 5–17 years of at least 60 min MVPA per day, and only two of 38 parents (5.3%) reached the recommended minimum level of moderate PA for adults, 150 min per week.

**Table 2:** Time spent in sedentary behaviour and physical activity categories of different intensities

Variable	Children (*n* = 39) Mean (SD) or *n* (%)	Parents (*n* = 38) Mean (SD) or *n* (%)	ICC (95% CI)	*p*-value	Mean paired difference (SD)	*p*-value
Child-parent pairs, *n*	39					
Accelerometer wear time, days^a^	6.3 (1.3)					
Reached recommended PA level^b^	6 (15.4)	2 (5.3)				
**SED** ^a^						
Time, minutes/day	588.8 (89.5)	736.4 (84.6)	**0.139 (0.086 to 0.400)**	**0.017**	-**147.6 (100.4)**	**< 0.001**
	min 406.8; max 745.9	min 593.0; max 950.7				
**LPA** ^a^						
Time, minutes/day	233.4 (60.4)	202.7 (60.7)	0.237 (-0.049 to 0.498)	0.050	**30.7 (73.5)**	**0.013**
	min 116.0; max 363.6	min 75.5; max 386.3				
**MVPA** ^a^						
Time, minutes/day	44.2 (19.9)	16.9 (17.9)	0.111 (-0.086 to 0.344)	0.081	**27.3 (23.6)** ^**c**^	**< 0.001**
	min 7.9; max 106.5	min 1.0; max 90.9				

ICC = Intra-class correlation coefficient; CI = confidence interval; LPA = Low physical activity; MPA = Moderate physical activity; MVPA = Moderate-to-vigorous-physical-activity; PA = Physical activity; SED = sedentary behaviour; SD = standard deviation; min = minimum value; max = maximum value

Values are given as ^a^ mean (standard deviation) or as ^b^ number (percentage)

^c^Wilcoxon’s signed rank statistic (SE)

Significant correlations and differences are indicated in bold

**Fig. 3:**
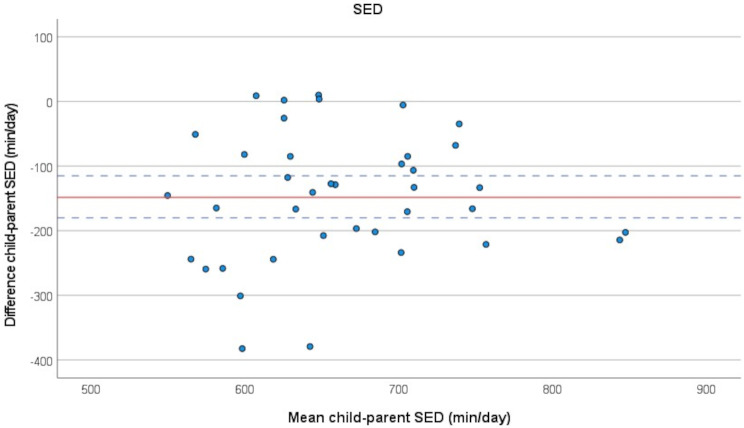
Bland-Altman plot illustrating difference between child and parent in minutes/day spent in sedentary time (SED), *n* = 39 pairs. Red solid line represents the mean of the within-pair difference, blue dotted lines represent the limits of agreement

**Fig. 4:**
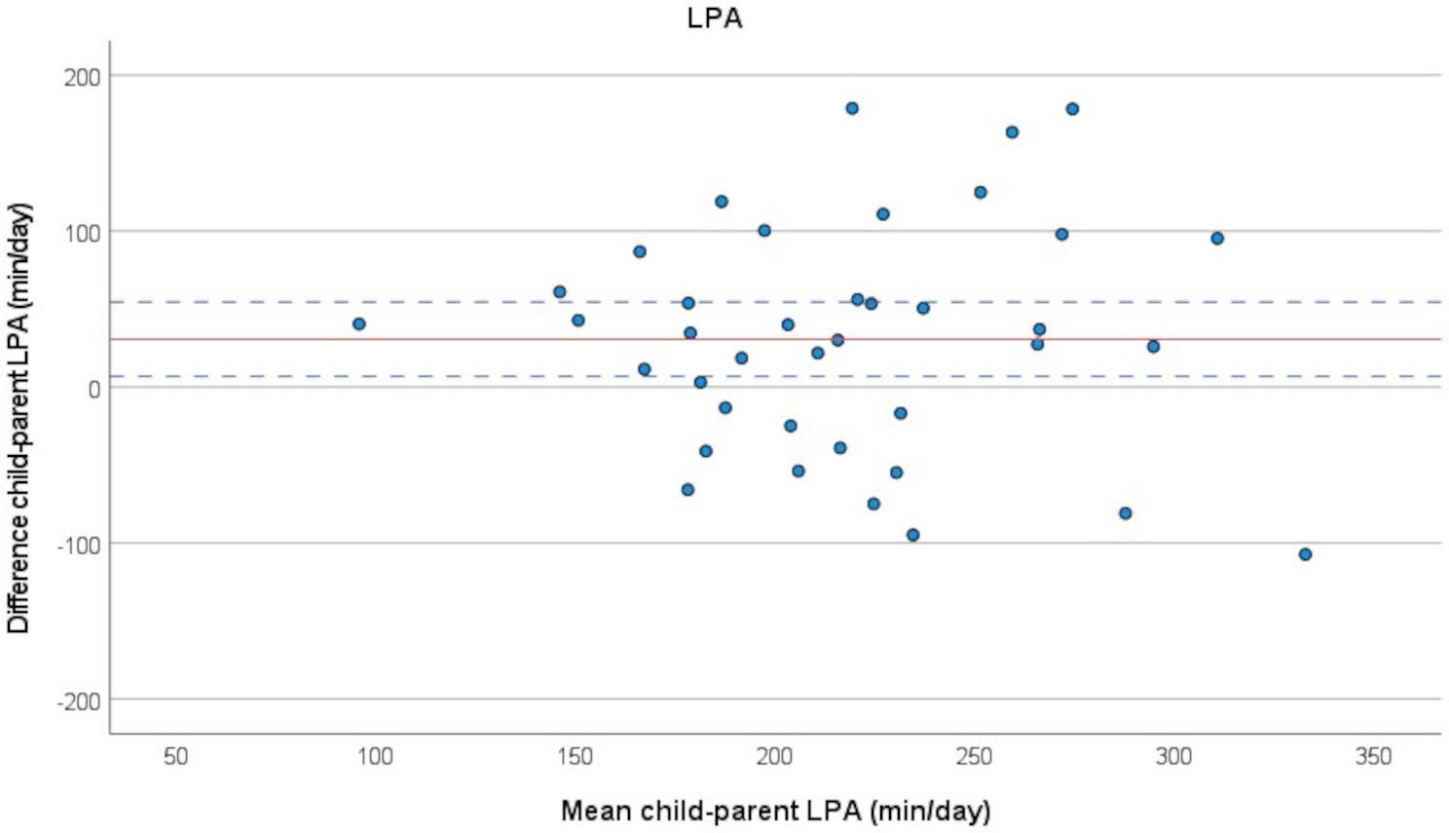
Bland-Altman plot illustrating difference between child and parent in minutes/day spent in low physical activity (LPA), *n* = 39 pairs. Red solid line represents the mean of the within-pair difference, blue dotted lines represent the limits of agreement

**Fig. 5:**
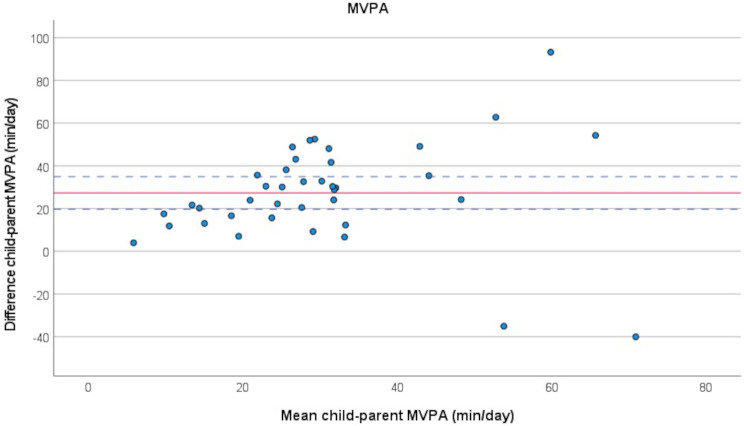
Bland-Altman plot illustrating difference between child and parent in minutes/day spent in moderate-to-vigorous physical activity (MVPA), *n* = 39 pairs. Red solid line represents the mean of the within-pair difference, blue dotted lines represent the limits of agreement

The results of the paired t-tests were further visualised in Bland-Altman plots, Figs. 3, 4 and 5. Systematic negative differences within child-parent pairs on the Y-axis mean parents in general spent more time in the category, which was the case for SED (Fig. 3), while positive differences within pairs mean children in general spent more time in the category, which was the case for LPA (Fig. 4) and MVPA (Fig. 5). No association between the difference between child and parent and the time spent in SED, LPA or MVPA was visible, but there was a large variation amongst child-parent pairs.

## Discussion

This study explored PA and SED in 39 school-aged children with obesity and one of their parents, and possible associations between them. Measures of PA and SED were collected using accelerometers. Main findings are that both children and parents spent most of the day in SED rather than being physically active and that only a small portion of both children and parents reached the recommended minimum time spent in MVPA. We found weak positive correlations between child and parent time spent in SED, LPA and MVPA; however, the correlation was only significant for SED. Children spent significantly more time than parents in both LPA and MVPA and parents spent more time in SED. The proportion who reached current PA guideline recommendations was small in both children and parents. Large variations were observed in how child and parent SED and PA were related.

Children not reaching the recommended time spent in MVPA is not surprising, as one of the inclusion criteria was to not be sufficiently physically active according to recommendations. It is also in line with a systematic review showing time spent in MVPA by children with obesity to be consistently below recommendations [44]. Earlier research has shown that few school-aged children in Sweden, regardless of weight status, reach the recommended PA levels for MVPA [45, 46]. Swedish data from the Generation PEP report 2024 reveal that only 2 of 10 children meet the PA recommendation of 60 min per day, with gender, age, and parental PA level and income being linked to how much children move [47]. The report shows that children whose parents are not physically active are less likely to meet the recommendation compared to children with more active parents, underscoring the important role of parents as models and the importance of involving them in behaviour change interventions targeting children.

Our findings contradict previous studies [20–22, 48, 49] showing positive, weak to moderate, associations between child and parent PA. A large meta-analysis of healthy children aged 2–18 years, reported a small effect of parental modelling on child PA [48]. However, the earlier reviews struggle with studies using heterogenic designs and often being based on self-report measures, and few of the studies focused on children with obesity.

Children with obesity have been reported to be less physically active and more sedentary than non-obese children [50], but study findings are inconsistent [51]. Liszewska et al. [52] and Kaseva et al. [53] both found that parental modelling by engaging in own PA predicted children’s PA regardless of the child’s BMI, in a Polish and Finnish population, respectively. Both studies used self-report which might have biased their findings. Our study found no statistically significant correlations between accelerometer-measured child and parent PA, although this may reflect the small sample size. The near-significance of the correlation observed in the LPA category suggests that a larger sample might reveal a statistically significant relationship.

A recent longitudinal study found a significant association between accelerometer-measured maternal and child PA in early childhood, but not between paternal and child PA, with no differences related to weight status [51]. Since MVPA is known to be the more important intensity category for health effects [42], it would also be an important intensity category for parents, to be able to influence their child’s behaviour. Ideally, it would be beneficial to replace time spent in SED with MVPA. For this patient group, however, even just replacing SED with LPA would be an important first step to increased PA and better health even though higher intensity PA is required to achieve health effects. Time spent in SED was in our study greater for both children and parents than in a recent study of healthy school-aged children aged 3–10 years and their parents in the Czech Republic [54], but only somewhat higher than a representative Swedish sample of 11–12 years old children [45].

Some of the differences between studies can be attributed to different accelerometers and data processing methods used to assess PA. Further, a lower cut-point for MVPA has been applied in previous research, mostly based on the 3 METs definition. However, the most important difference is probably that previous studies included younger children (age range 2–10 years) compared to our study (age range 6–12 years), where younger children may be more dependent of their parents [51, 55, 56], with more associated PA.

### Study limitations and strengths

The main limitations of this study are the cross-sectional design, preventing us from drawing any conclusions on causal relationships, and the small sample size. As the study was part of a subsequent feasibility study, no power calculation was conducted. Our initial sample size target was 60 children, which we did not reach due to unforeseen circumstances, e.g. staffing problems related to the COVID-19 pandemic. A larger sample size could have enabled the detection of additional significant correlations. Another potential limitation is our choice of defining a valid week as containing only two valid days. This allowed us to increase our sample size while a higher number of valid days may have provided more reliable data. However, we controlled the results with sensitivity analysis and feel confident in the reliability of our findings.

Comparison of PA between children and adults is still hampered by the challenge to develop measures that are directly comparable between age groups [30]. Calibration studies usually relate acceleration data that are biomechanical in nature to energy expenditure that depends on the individual metabolism. Both variables change across childhood. Today, there are no directly comparable measures of PA. Our determination of cut-point for MVPA was based on a combination of lab calibration data and free-living associations with cardiometabolic health, but is not optimal. Consequently, there is a need for future research to establish measures of PA directly comparable between age groups, to enable more reliable comparisons of PA patterns between children and their parents. Examining parent-child correlations across the 24-hour movement spectrum would also be an important direction for future research, using methods specifically designed to capture and analyse sleep outcomes alongside physical activity data.

The findings are potentially affected by selection bias in that we used a self-selected, non-random sample. Children and parents who have positive attitudes towards PA and are more physically active might be the ones accepting invitations to this kind of research project.

We did not collect information on parents’ BMI. Since there is a genetic factor to obesity, some of the parents likely had a high BMI as well, which may have affected their PA level. Neither did we ask for information on family living arrangements; if parents were divorced, we do not know which parent the child lived with during the week of PA measurement. In case the child lived with the non-participating parent during the measurement, that parent might have been more important for the child’s PA.

The main strengths of the study are our use of accelerometer-measured data and the concurrent PA measures of both child and parent. Further, we used an improved accelerometer processing method that better captures PA across the intensity range in both children and adults [34]. We recruited participants over a long period that covered all seasons. Both season and weather are known to affect PA behaviour [24], and while we could not do much about the weather, our recruiting in all seasons likely minimised the seasonal effect on PA.

## Conclusions and implications

The findings of this study indicate a significant but weak association between children’s and parents’ SED in this sample of 6–12-year-old children with obesity. Although the association in LPA was stronger, it did not reach statistical significance. No association was found in MVPA. The large variations in differences between child and parent could suggest that other components of parental involvement than role modelling through own physical activity, might be of greater importance in PA interventions. For example, parents could support their children to be physically active by facilitating availability and access to PA opportunities, initiating activities, and offering encouragement. Generalisability of our findings is likely to be limited, and more research is needed– in larger samples, other settings, and using longitudinal designs– to better understand potential links between PA in children with obesity and PA of their parents.

Our findings may be useful in guiding adaptations of PA interventions, such as the Swedish PAP model, for children with obesity. The significant association in SED implies that this particular behaviour potentially has some role model importance. Perhaps the emphasis of a PAP intervention for this population should initially be on reducing sedentary time, a behavioural change that over time will enable increased PA. Although no significant correlations were found between children’s and their parents’ time spent in PA, differences in MVPA and LPA were small in some child-parent pairs. This finding suggests that some children could benefit from their parents being involved in interventions as role models through their own PA behaviours, while others may not. This would need to be explored on an individual level, which is consistent with the family-centred approach offered in the PAP model. To design sustainable PA interventions for this group, future research should further explore children’s and parents’ own experiences and needs.

## Electronic supplementary material

Below is the link to the electronic supplementary material.

Supplementary Material 1

## Data Availability

The datasets used and/or analysed in the current study are available from the corresponding author on reasonable request.

## Abbreviations

LPA: Low intensity physical activity
MVPA: Moderate-to-vigorous physical activity
PA: Physical activity
SED: Sedentary behaviour

## References

[1] GBD 2015 Risk Factors Collaborators. Global, regional, and national comparative risk assessment of 79 behavioural, environmental and occupational, and metabolic risks or clusters of risks, 1990–2015: a systematic analysis for the global burden of disease study 2015. Lancet. 2016;388(10053):1659–724. 27733284 10.1016/S0140-6736(16)31679-8 PMC5388856

[2] Abarca-Gómez L, Abdeen ZA, Hamid ZA, Abu-Rmeileh NM, Acosta-Cazares B, Acuin C, et al. Worldwide trends in body-mass index, underweight, overweight, and obesity from 1975 to 2016: a pooled analysis of 2416 population-based measurement studies in 128·9 million children, adolescents, and adults. Lancet. 2017;390(10113):2627–42. 29029897 10.1016/S0140-6736(17)32129-3 PMC5735219

[3] Simmonds M, Llewellyn A, Owen CG, Woolacott N. Predicting adult obesity from childhood obesity: a systematic review and meta-analysis. Obes Rev. 2016;17(2):95–107. 26696565 10.1111/obr.12334

[4] Ward ZJ, Long MW, Resch SC, Giles CM, Cradock AL, Gortmaker SL. Simulation of growth trajectories of childhood obesity into adulthood. N Engl J Med. 2017;377(22):2145–53. 29171811 10.1056/NEJMoa1703860 PMC9036858

[5] Public Health Agency of Sweden. Overweight and obesity are common and increase with age in 6–9 year olds [internet] Stockholm: Public Health Agency of Sweden; 2021 [cited 19 Sept 2024]. Available from: https://www.folkhalsomyndigheten.se/publikationer-och-material/publikationsarkiv/o/overweight-and-obesity-are-common-and-increase-with-age-in-69-year-olds/

[6] Jebeile H, Kelly AS, O’Malley G, Baur LA. Obesity in children and adolescents: Epidemiology, causes, assessment, and management. Lancet Diabetes Endocrinol. 2022;10(5):351–65. 35248172 10.1016/S2213-8587(22)00047-X PMC9831747

[7] Kumar S, Kelly AS. Review of childhood obesity: From epidemiology, etiology, and comorbidities to clinical assessment and treatment. Mayo Clin Proc. 2017;92(2):251– 65. 10.1016/j.mayocp.2016.09.017 28065514

[8] Headid Iii RJ, Park SY. The impacts of exercise on pediatric obesity. Clin Expr Pediatr. 2021;64(5):196–207. 10.3345/cep.2020.00997 PMC8103043 32777917

[9] Lauby-Secretan B, Scoccianti C, Loomis D, Grosse Y, Bianchini F, Straif K. Body fatness and cancer–viewpoint of the IARC working group. N Engl J Med. 2016;375(8):794–8. 27557308 10.1056/NEJMsr1606602 PMC6754861

[10] Fontaine KR, Redden DT, Wang C, Westfall AO, Allison DB. Years of life lost due to obesity. JAMA. 2003;289(2):187–93. 12517229 10.1001/jama.289.2.187

[11] World Health Organization. Report on the commission on ending childhood obesity [internet]. Geneva: World Health Organization. 2016 [cited 19 Sept 2024]. Available from: https://www.who.int/publications/i/item/9789241510066

[12] Wolfenden L, Barnes C, Jones J, Finch M, Wyse RJ, Kingsland M, et al. Strategies to improve the implementation of healthy eating, physical activity and obesity prevention policies, practices or programmes within childcare services. Cochrane Database Syst Rev. 2020;2(2):CD011779. 32036618 10.1002/14651858.CD011779.pub3 PMC7008062

[13] Berg U, Ekblom Ö, Onerup A. Recommendations on physical activity and sedentary behaviour for children and youth. In: Dohrn I-M, Jansson E, Börjesson M, Hagströmer M, editors. Physical activity in the prevention and treatment of disease, FYSS 2021. Stockholm: Läkartidningen Förlag AB; 2021.

[14] Janssen I, Leblanc AG. Systematic review of the health benefits of physical activity and fitness in school-aged children and youth. Int J Behav Nutr Phys Act. 2010;7:40. 20459784 10.1186/1479-5868-7-40 PMC2885312

[15] Montero D, Walther G, Perez-Martin A, Roche E, Vinet A. Endothelial dysfunction, inflammation, and oxidative stress in obese children and adolescents: markers and effect of lifestyle intervention. Obes Rev. 2012;13(5):441–55. 22133012 10.1111/j.1467-789X.2011.00956.x

[16] Mead E, Brown T, Rees K, Azevedo LB, Whittaker V, Jones D, et al. Diet, physical activity and behavioural interventions for the treatment of overweight or obese children from the age of 6 to 11 years. Cochrane Database Syst Rev. 2017;6:CD012651. 28639319 10.1002/14651858.CD012651 PMC6481885

[17] Morgan EH, Schoonees A, Sriram U, Faure M, Seguin-Fowler RA. Caregiver involvement in interventions for improving children’s dietary intake and physical activity behaviors. Cochrane Database Syst Rev. 2020;1(1):CD012547. 31902132 10.1002/14651858.CD012547.pub2 PMC6956675

[18] Tate EB, Shah A, Jones M, Pentz MA, Liao Y, Dunton G. Toward a better understanding of the link between parent and child physical activity levels: the moderating role of parental encouragement. J Phys Act Health. 2015;12(9):1238–44. 25494399 10.1123/jpah.2014-0126 PMC5504529

[19] McAlister AL, Perry CL, Parcel GS. How individuals, environments, and health behaviors interact. In: Glanz K, Rimer BK, Viswanath K, editors. Health behavior and health education: theory, research, and practice. Hoboken: Wiley; 2008.

[20] Matos R, Monteiro D, Amaro N, Antunes R, Coelho L, Mendes D et al. Parents’ and children’s (6–12 years old) physical activity association: A systematic review from 2001 to 2020. Int J Environ Res Public Health. 2021;18(23). 10.3390/ijerph182312651 PMC8656881 34886372

[21] Petersen TL, Møller LB, Brønd JC, Jepsen R, Grøntved A. Association between parent and child physical activity: a systematic review. Int J Behav Nutr Phys Act. 2020;17(1):67. 32423407 10.1186/s12966-020-00966-z PMC7236180

[22] McMurray RG, Berry DC, Schwartz TA, Hall EG, Neal MN, Li S, et al. Relationships of physical activity and sedentary time in obese parent-child dyads: a cross-sectional study. BMC Public Health. 2016;16:124. 26851940 10.1186/s12889-016-2795-5 PMC4744403

[23] Migueles JH, Cadenas-Sanchez C, Ekelund U, Delisle Nystrom C, Mora-Gonzalez J, Lof M, et al. Accelerometer data collection and processing criteria to assess physical activity and other outcomes: a systematic review and practical considerations. Sports Med (Auckland NZ). 2017;47(9):1821–45. 10.1007/s40279-017-0716-0 PMC6231536 28303543

[24] Arvidsson D, Fridolfsson J, Börjesson M. Measurement of physical activity in clinical practice using accelerometers. J Intern Med. 2019;286(2):137–53. 30993807 10.1111/joim.12908

[25] Trost SG, Kerr LM, Ward DS, Pate RR. Physical activity and determinants of physical activity in obese and non-obese children. Int J Obes Relat Metab Disord. 2001;25(6):822–9. 10.1038/sj.ijo.0801621 11439296 10.1038/sj.ijo.0801621

[26] Chan KM, Rahem SM, Teo HO, Curcio J, Mushiyev S, Faillace R, Bochner R, Bargman R, Farbod Raiszadeh F. Understanding family dynamics of obesity: do parents and children lose and gain weight together? Pediatr Obes. 2024;19(6):e13097. 10.1111/ijpo.13097. Epub 2024 Apr 7. 38583983 10.1111/ijpo.13097

[27] Blanchet R, Kengneson CC, Bodnaruc AM, Gunter A, Giroux I. Factors influencing parents’ and children’s misperception of children’s weight status: a systematic review of current research. Curr Obes Rep. 2019;8(4):373–412. 10.1007/s13679-019-00361-1 31701349 10.1007/s13679-019-00361-1

[28] Paul TK, Sciacca RR, Bier M, Rodriguez J, Song S, Giardina EG. Size misperception among overweight and obese families. J Gen Intern Med. 2015;30(1):43–50. 10.1007/s11606-014-3002-y. Epub 2014 Sep 16. 25223750 10.1007/s11606-014-3002-y PMC4284259

[29] Notara V, Giannakopoulou SP, Sakellari E, Panagiotakos DB. Family-related characteristics and childhood obesity: a systematic literature review. Int J Caring Sci. 2020;13(1):61.

[30] Wentz E, Björk A, Dahlgren J. Neurodevelopmental disorders are highly over-represented in children with obesity: a cross-sectional study. Obesity. 2017;25(1):178–84. 27874270 10.1002/oby.21693

[31] Dellenmark-Blom M, Järvholm K, Sjögren L, Levinsson A, Dahlgren J. Neurodevelopmental disorders in children seeking obesity treatment- associations with intellectual ability and psychiatric conditions. Front Psychiatry. 2024;15:1332598. 39224476 10.3389/fpsyt.2024.1332598 PMC11366696

[32] Bernhardsson S, Boman C, Lundqvist S, Arvidsson D, Börjesson M, Larsson MEH, et al. Implementation of physical activity on prescription for children with obesity in paediatric health care (IMPA): protocol for a feasibility and evaluation study using quantitative and qualitative methods. Pilot Feasibil Stud. 2022;8(1):117. 10.1186/s40814-022-01075-3 PMC9158137 35650617

[33] Clark CCT, Nobre GC, Fernandes JFT, Moran J, Drury B, Mannini A, Gronek P, Podstawski R. Physical activity characterization: does one site fit all? Physiol. Meas. 2018;39(9). 10.1088/1361-6579/aadad0 30113317

[34] Fridolfsson J, Börjesson M, Buck C, Ekblom Ö, Ekblom-Bak E, Hunsberger M et al. Effects of frequency filtering on intensity and noise in accelerometer-based physical activity measurements. Sensors. 2019;19(9). 10.3390/s19092186 PMC6539652 31083538

[35] Fridolfsson J, Börjesson M, Arvidsson D. A biomechanical re-examination of physical activity measurement with accelerometers. Sensors. 2018;18(10). 10.3390/s18103399 PMC6210008 30314272

[36] World Health Organization. WHO guidelines on physical activity and sedentary behaviour. Geneva: World Health Organization; 2020. 33369898

[37] Arvidsson D, Fridolfsson J, Buck C, Ekblom O, Ekblom-Bak E, Lissner L et al. Reexamination of accelerometer calibration with energy expenditure as criterion: Vo(2net) instead of MET for age-equivalent physical activity intensity. Sensors. 2019;19(15). 10.3390/s19153377 PMC6695745 31374854

[38] Butte NF, Watson KB, Ridley K, Zakeri IF, McMurray RG, Pfeiffer KA, et al. A youth compendium of physical activities: activity codes and metabolic intensities. Med Sci Sports Exerc. 2018;50(2):246–56. 28938248 10.1249/MSS.0000000000001430 PMC5768467

[39] Herrmann SD, Willis EA, Ainsworth BE, Barreira TV, Hastert M, Kracht CL, et al. 2024 adult compendium of physical activities: a third update of the energy costs of human activities. J Sport Health Sci. 2024;13(1):6–12. 38242596 10.1016/j.jshs.2023.10.010 PMC10818145

[40] Fridolfsson J, Buck C, Hunsberger M, Baran J, Lauria F, Molnar D, et al. High-intensity activity is more strongly associated with metabolic health in children compared to sedentary time: a cross-sectional study of the i.Family cohort. Int J Behav Nutr Phys Act. 2021;18(1):90. 34229708 10.1186/s12966-021-01156-1 PMC8261968

[41] Fridolfsson J, Ekblom-Bak E, Ekblom O, Bergstrom G, Arvidsson D, Borjesson M. Fitness-related physical activity intensity explains most of the association between accelerometer data and cardiometabolic health in persons 50–64 years old. Br J Sports Med. 2024. 10.1136/bjsports-2023-107451 PMC11671887 38997147

[42] Karlberg J, Luo ZC, Albertsson-Wikland K. Body mass index reference values (mean and SD) for Swedish children. Acta Paediatr. 2001;90(12):1427–34. 11853342 10.1111/j.1651-2227.2001.tb01609.x

[43] Koo TK, Li MY. A guideline of selecting and reporting intraclass correlation coefficients for reliability research. J Chiropr Med. 2016;15(2):155–63. 27330520 10.1016/j.jcm.2016.02.012 PMC4913118

[44] Elmesmari R, Martin A, Reilly JJ, Paton JY. Comparison of accelerometer measured levels of physical activity and sedentary time between obese and non-obese children and adolescents: a systematic review. BMC Pediatr. 2018;18(1):106. 29523101 10.1186/s12887-018-1031-0 PMC5844092

[45] Nyberg G, Kjellenberg K, Fröberg A, Lindroos AK. A national survey showed low levels of physical activity in a representative sample of Swedish adolescents. Acta Paediatr. 2020;109(11):2342–53. 32266736 10.1111/apa.15251

[46] Folkhälsomyndigheten. Barns och ungas rörelsemönster. Resultat från objektivt uppmätt fysisk aktivitet, skolbarns hälsovanor 2017/2018. Stockholm: 2019.

[47] Annwall E, Ersberg L, J-son Höök M. The PEP report 2024: Economic inequality contributes to the children’s health gap. Stockholm: 2024.

[48] Yao CA, Rhodes RE. Parental correlates in child and adolescent physical activity: a meta-analysis. Int J Behav Nutr Phys Act. 2015;12:10. 25890040 10.1186/s12966-015-0163-y PMC4363182

[49] Su DLY, Tang TCW, Chung JSK, Lee ASY, Capio CM, Chan DKC. Parental influence on child and adolescent physical activity level: a meta-analysis. Int J Behav Nutr Phys Act. 2022;19(24). 10.3390/ijerph192416861 PMC9778652 36554746

[50] Danielsen YS, Skjåkødegård HF, Mongstad M, Hystad SW, Olsson SJG, Kleppe M, et al. Objectively measured physical activity among treatment seeking children and adolescents with severe obesity and normal weight peers. Obes Sci Pract. 2022;8(6):801–10. 36483122 10.1002/osp4.624 PMC9722458

[51] Bergqvist-Noren L, Hagman E, Xiu L, Marcus C, Hagstromer M. Physical activity in early childhood: a five-year longitudinal analysis of patterns and correlates. Int J Behav Nutr Phys Act. 2022;19(1):47. 35443696 10.1186/s12966-022-01289-x PMC9022334

[52] Liszewska N, Scholz U, Radtke T, Horodyska K, Liszewski M, Luszczynska A. Association between children’s physical activity and parental practices enhancing children’s physical activity: the moderating effects of children’s BMI z-score. Front Psychol. 2017;8:2359. 29422877 10.3389/fpsyg.2017.02359 PMC5789260

[53] Kaseva K, Hintsa T, Lipsanen J, Pulkki-Råback L, Hintsanen M, Yang X, et al. Parental physical activity associates with offspring’s physical activity until middle age: a 30-year study. J Phys Act Health. 2017;14(7):520–31. 28290745 10.1123/jpah.2016-0466

[54] Sigmundova D, Voracova J, Dygryn J, Vorlicek M, Sigmund E. Parent-child associations in accelerometer-measured physical activity and sedentary behaviour: the Famipass study. Children. 2024;11(6). 10.3390/children11060710 PMC11202232 38929289

[55] Sigmundová D, Sigmund E, Badura P, Hollein T. Parent-child physical activity association in families with 4-to 16-year-old children. Int J Environ Res Public Health. 2020;17(11). 10.3390/ijerph17114015 PMC7312858 32516925

[56] Julius BR, O’Shea AMJ, Francis SL, Janz KF, Laroche H. Leading by example: Association between mother and child objectively measured physical activity and sedentary behavior. Pediatr Exerc Sci. 2021;33(2):49–60. 33819915 10.1123/pes.2020-0058 PMC8845373

